# Increased capture of *Aedes aegypti* (Linnaeus, 1762) (Diptera: Culicidae) by removing one ADULTRAP component

**DOI:** 10.1590/0037-8682-0043-2019

**Published:** 2020-01-27

**Authors:** Noemí Silva Ferreira, Gilson Correia de Carvalho, Yasmin Gabriela Alves dos Santos, Adriano Figueiredo Monte-Alegre

**Affiliations:** 1 Graduada em Medicina Veterinária, Universidade Federal da Bahia, Salvador, BA, Brasil.; 2 Universidade Federal da Bahia, Departamento de Biotecnologia, Instituto de Ciências da Saúde, Salvador, BA, Brasil.; 3 Programa de Pós-Graduação em Ecologia: Teoria, Aplicação e Valores - PPGECOTAV, Instituto de Biologia, Universidade Federal da Bahia, Salvador, BA, Brasil.; 4 Programa de Pós-Graduação em Ecologia - PPGECO, Instituto de Biologia , Universidade Federal da Bahia, Salvador, BA, Brasil.; 5 Graduada em Biotecnologia, Universidade Federal da Bahia, Salvador, BA, Brasil.; 6 Universidade Federal da Bahia, Departamento de Biointeração, Instituto de Ciências da Saúde, Salvador, BA, Brasil.

**Keywords:** Adultrap®, Aedes aegypti, Insect attractiveness, Insect traps

## Abstract

**INTRODUCTION:**

*Aedes aegypti* is the main vector responsible for the transmission of numerous arboviruses. Adultrap® has been developed to catch these insects.

**METHODS:**

We tested the effectiveness of capturing adults with and without one of the components of Adultrap®.

**RESULTS:**

The mean number of insects caught by the original trap was 1.25 (standard deviation = 1.28), while the average obtained with the modified trap was 8.88 (standard deviation = 3.44). The medians were statistically different (p = 0.001) according to the Mann-Whitney test.

**CONCLUSIONS:**

The modification of Adultrap® increased the average catch of *Ae. aegypti* by up to seven times.

*Aedes aegypti* was introduced to Brazil during the colonial period and is now distributed across its 27 federative units in more than 3,587 municipalities^1^. The species is predominantly urban and its anthropophilic behavior is implicated in the transmission of the four serotypes of dengue virus (DENV1-4), in the transmission of urban yellow fever virus (YFV), and other arboviruses such as Chikungunya (CHIKV) and Zika virus (ZIKV)^2^. In 2014, ZIKV was isolated in Brazil and, in the same year, 62 suspected cases of CHIKV were reported in Salvador-BA^3^. Studies of insect vectors frequently start with their capture in the field and/or in urban areas. The choice of trap is therefore critical to the success of capture and subsequent analysis. Having the specificity to attract many specimens of the target species, while maintaining the organism’s integrity are features of an effective entomological trap. When these criteria are met, the trap is an essential tool in the field of medical entomology. Currently, many traps are sold in various sizes with different mechanisms for capturing insect vectors carrying pathogens^4^ ^,^ ^5^ ^,^ ^6^ ^,^ ^7^ ^,^ ^8^. The Adultrap®^5^ is a trap commercialized to capture mosquitoes without killing them. The mechanism used in Adultrap® is based on behavioral and physiological characteristics of adult mosquitoes. The trap is made of plastic and is dark in color, which creates an attractive environment for them. In a study by Gomes et al.,^5^ the effectiveness of Adultrap® was confirmed, especially for the capture of *Aedes aegypti* adults. Furthermore, the container structure, its color, presence of water, and odor, among other factors, are of great importance for successfully attracting and capturing adult *Aedes* insects^9^. Any variation in these elements might alter the effectiveness of the capture mechanism. Based on the analysis carried out on the components present in commercial Adultrap® units, we verified that the component that closes the container prevents direct visualization of the water inside. The Water Insulation Plate (WIP) is made of a microporous material that allows water vapor to pass through, which can be sensed by the insect. To assess whether closing the container using the WIP reduces the yield of the catch, we compared the effectiveness of Adultraps with and without the WIP.

This study was conducted in the area surrounding the Institute of Health Sciences (ICS) at the Federal University of Bahia and the School of Medicine (FAMED), both located at Vale do Canela in Salvador-BA-Brazil. The coordinates of the sample points were obtained using a GPS *(Global Positioning System)* with a projected coordinate Universal Transverse Mercator (UTM) and South American Datum-69 (SAD-69) as the reference datum (Figure 1).

**FIGURE 1:**
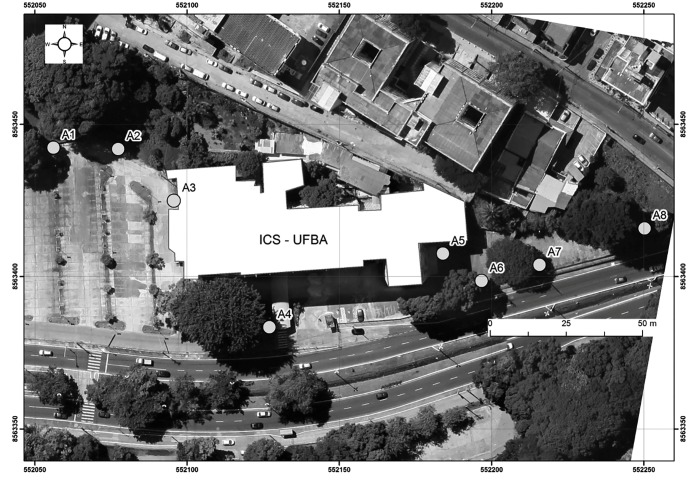
Location of sample units (A1-A8).

Adultrap® is composed of a concave component with a fixing point at a diameter of 24 cm (A). The central part has an opening of 7 cm in diameter with one edge 4 cm in height, which faces the concave component (A) and serves as a connection to part (B). It is constructed by assembling the four transparent cones (C) with central holes of 1.0 cm in diameter to the sides of the trap. A thin screen with 54 apertures/cm^2^ (D) isolates the water or the attractive bait trap body, separating parts (B) and (E). The container (water vat) that collects water (E) has a diameter of 14.5 cm and a base of 8.5 cm and can hold up to 600 ml of water (Figure 2)*.* The sides of the trap are composed of four plates formed out of the same screen measuring 15 cm long and 10.5 cm wide, fixed to columns of rigid plastic (F). The tiny holes allow the passage of air and a certain degree of natural light (F). The diameter of this component measures 24 cm, completing the body of the trap. After assembling and mounting all the parts, two compartments are formed (Figure 2). The first is the opening for mosquitoes to enter and the second traps them between the cones and the screen wall. An external strap fixes it in place and allows it to be carried by hand to the laboratory^4^.

**FIGURE 2:**
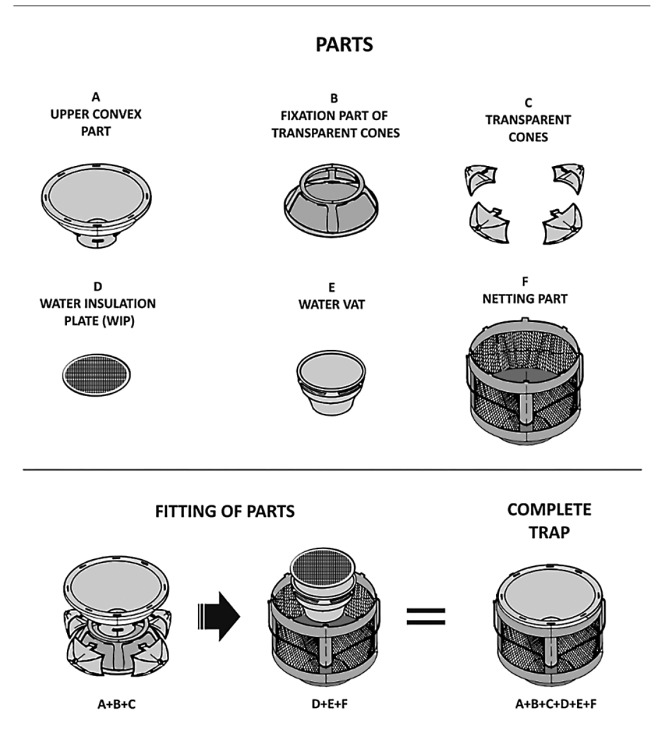
Diagram of the Adultrap® showing each component and how the parts are mounted into one trap.

In order to assess any differences between the original Adultrap® units and those with the modification (removal of the component D, as cited above) for effectiveness in capturing mosquitoes, Adultrap® traps with and without component D were distributed at eight different points located around ICS and FAMED (Figure 1). All traps were prepared as suggested in Donatti and Gomes^4^, by placing tap water in compartment E (the water vat). The only change introduced to the experimental group of Adultraps was the withdrawal of the WIP. The experiment was conducted between July 2014 and October 2014. Over these four months, the traps were placed around ICS and FAMED at the points A1, A2, A3, A4, A5, A6, A7, and A8, according to the map in Figure 1. Two forms of the traps’ dispositions in the field were adopted to avoid biases in the experiment. During the first period (July and August), Adultraps with the WIP (control group) were placed at points A1, A2, A3, and A4 and Adultraps without the WIP (experimental group) were placed at points A5, A6, A7, and A8. After a week, the locations were switched. This pattern was repeated week by week for two months. During the second period (September and October), the process was altered. The Adultraps with the WIP (control group) were placed at all points (A1-A8) for a week and then replaced by the Adultraps without the WIP (experimental group) for another week. This was repeated week by week for another two months.

The traps were inspected daily. The insects captured were identified and recorded. The integrity of the traps and their functioning were evaluated during these inspections. At the end of each week, the traps without the WIP (D) were removed, and the water vat (E) was cleaned with sponge and water to remove any eggs laid by insects on the walls of the container. Only insects of the species *Aedes aegypti* (adults) were counted and other species were discarded. 

The data for all periods were recorded for each sample point and treatment group. Data were assessed for normality and homogeneity of variance. A Shapiro-Wilk test was used to evaluate normality, and a Bartlett test was used to evaluate homogeneity of variance. The Shapiro-Wilk test showed that both sets of data on capture rate (with and without the insulating screen) failed to reject the null hypothesis of normality (p > 0.05). However, the null hypothesis of homogeneity of variances was rejected after applying Bartlett’s test (p = 0.019). Therefore, to compare catches between traps (with and without the WIP), a Mann-Whitney nonparametric test was used. All statistical analyses and the creation of a box plot were performed using the statistical package R version 3.2.3^10^. The level of significance considered for all tests was 0.05.

During the experimental period, a total of 81 *Aedes aegypti* adults were sampled, 10 of which were captured in traps with the original marketed structure and 71 that were captured with the modified trap. The average number of specimens captured with the original trap was 1.25 (with a standard deviation of 1.28), while the average number captured with the modified trap was 8.88 (with a deviation of 3.44), representing a seven-fold increase of average capture for the trap without the WIP component. Figure 3 shows a box plot (with the median, 1st and 3rd quartiles, and amplitude of catch data plotted) of abundance data, grouped by the two traps used in this study (with the WIP and without the WIP). The difference in catch abundance between the two treatments was significant, indicating that modifying the trap induced a difference greater than what is expected by chance (p = 0.001). The results demonstrate a significant difference in the capture of *Aedes aegypti* between Adultraps without component D (WIP) compared to those with the WIP.

**FIGURE 3:**
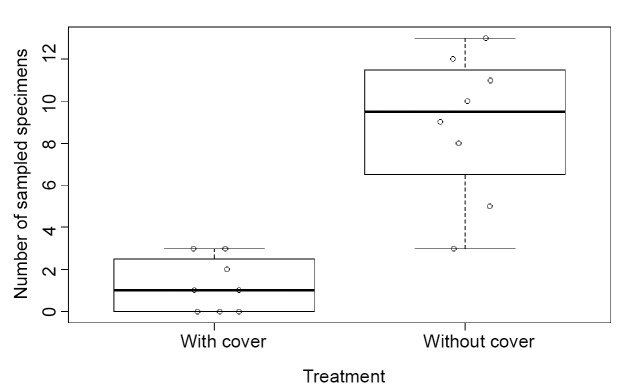
The number of sampled specimens of *Aedes aegypti* caught using Adultrap® with and without the WIP; the box plot shows the median, 1st-3rd quartile, and maximum and minimum values.

Studies have shown that visual perception of the environment plays a fundamental role in the actions of many insects. Mosquitoes of the species *Anopheles gambiae* and *Aedes aegypti* have retinal photoreceptors that allow them to be more sensitive to long and short wavelengths^11^ ^,^ ^12^. One of the most studied photoreceptors is the rhodopsin protein, which can be found in vertebrates and invertebrates. *Aedes aegypti* expresses this protein upon absorption of UV wavelengths (lengths shorter than 400 nm)^11^ ^,^ ^13^. Water can reflect UV rays, and the use of these photoreceptors could allow mosquitoes to detect appropriate waterbodies for oviposition. Besides the visual photoreceptors, it has been shown that in the feelers of *Aedes aegypti,* there are sensilla, which have receptors for water vapor, carbon dioxide (CO_2_), and other compounds^14^ ^,^ ^15^. As the area of a body of water gets larger, a greater amount of water is evaporated. Therefore, the few insects captured in the original traps may be the result of a reduction in attractiveness caused by the water compartment being blocked by the WIP. Without the visual and sensory stimulation of the water, the trap has less influence over the behavior of *Aedes aegypti* less, reducing its attractiveness. The results of this study have shown that removing the WIP component from commercial Adultraps increases the capture of *Aedes aegypti* more than seven-fold. Sunlight reflected by the exposed water can easily be captured by the sensitive cells present in the compound eyes of the mosquitoes. By blocking the water from the insects’ view, this perception does not occur. This leads to a reduction in the potential attractiveness and the capture of the insects. It is likely that the WIP component of the Adultrap® was originally designed to prevent water from being exposed and becoming a breeding ground for the mosquitoes. However, considering that the time between the L1 stage of insects (larvae hatched) and the formation of the winged adult is approximately 10-12 days, this problem can be easily managed with frequent inspections. During periods of collection using the traps, they should be inspected at least every four days, eliminating the risk of successful breeding.

The comparison of the traps with and without the WIP has been well established in this study. However, testing it in different areas over a longer period could further consolidate these findings and assist in the discovery of other species of hematophagous culicids.

Data obtained in this study reinforce the theory that exposed water is the most attractive source for insects of the species *Aedes aegypti*, above the type and color of the trap container. In addition, the removal of component D (the WIP) from the Adultrap® should be used as a tool by researchers and those working for zoonoses control centers seeking to enhance the efficiency of *Aedes* catches using Adultraps and to strengthen control strategies for the benefit of public health through further studies.
